# Barriers and facilitators for implementing the WHO Safe Childbirth Checklist (SCC) in Mozambique: A qualitative study using the Consolidated Framework for Implementation Research (CFIR)

**DOI:** 10.1371/journal.pgph.0003174

**Published:** 2024-09-05

**Authors:** Anqi He, Elsa Luís Kanduma, Rafael Pérez-Escamilla, Devina Buckshee, Eusébio Chaquisse, Rosa Marlene Cuco, Mayur Mahesh Desai, Danícia Munguambe, Sakina Erika Reames, Isaías Ramiro Manuel, Donna Spiegelman, Dong Xu

**Affiliations:** 1 Department of Health Policy, Yale School of Public Health, New Haven, Connecticut, United States of America; 2 Comité para Saúde de Moçambique, Maputo City, Mozambique; 3 Mozambique Ministry of Health, Maputo City, Mozambique; 4 Department of Social and Behavioral Sciences, Yale School of Public Health, New Haven, Connecticut, United States of America; 5 Department of Chronic Disease Epidemiology, Yale School of Public Health, New Haven, Connecticut, United States of America; 6 Department of Epidemiology of Microbial Diseases, Yale School of Public Health, New Haven, Connecticut, United States of America; 7 Department of Biostatistics, Yale School of Public Health, New Haven, Connecticut, United States of America; 8 Department of Health Systems and Global Health, Southern Medical University, Guangzhou, Guangdong, China; PLOS: Public Library of Science, UNITED STATES OF AMERICA

## Abstract

High maternal and neonatal mortality rates persist in Mozambique, with stillbirths remaining understudied. Most maternal and neonatal deaths in the country are due to preventable and treatable childbirth-related complications that often occur in low-resource settings. The World Health Organization introduced the Safe Childbirth Checklist (SCC) in 2015 to reduce adverse birth outcomes. The SCC, a structured list of evidence-based practices, targets the main causes of maternal and neonatal deaths and stillbirths in healthcare facilities. The SCC has been tested in over 35 countries, demonstrating its ability to improve the quality of care. However, it has not been adopted in Mozambique. This study aimed to identify potential facilitators and barriers to SCC implementation from the perspective of birth attendants, clinical administrators, and decision-makers to inform future SCC implementation in Mozambique. We conducted a qualitative study involving focus group discussions with birth attendants (n = 24) and individual interviews with clinical administrators (n = 6) and decision-makers (n = 8). The Consolidated Framework for Implementation Research guided the questions used in the interviews and focus group discussions, as well as the subsequent data analysis. A deductive thematic analysis of Portuguese-to-English translated transcripts was performed. In Mozambique, most barriers to potential SCC implementation stem from the challenges within a weak health system, including underfunded maternal care, lack of infrastructure and human resources, and low provider motivation. The simplicity of the SCC and the commitment of healthcare providers to better childbirth practices, combined with their willingness to adopt the SCC, were identified as major facilitators. To improve the feasibility of SCC implementation and increase compatibility with current childbirth routines for birth attendants, the SCC should be tailored to context-specific needs. Future research should prioritize conducting pre-implementation assessments to align the SCC more effectively with local contexts and facilitate sustainable enhancements in childbirth practices.

## Introduction

The global efforts towards achieving the World Health Organization (WHO) Sustainable Development Goal 3 (Ensure healthy lives and promote well-being for all at all ages) have significantly reduced pregnancy-related deaths, especially in sub-Saharan Africa (SSA) [1], one of the regions most affected by maternal and neonatal mortality in the world [2]. Guided by the Mozambican Strategic Plan for Health Sector 2014–19 and the Government’s Five-Year Plan 2020–24 [3, 4], the Mozambican government has substantially improved maternal and child health (MCH) outcomes by expanding care services and enhancing their quality. Between 2015 and 2021, maternal mortality in Mozambique decreased by 75.8% [5], neonatal mortality by 8% [6], and stillbirth rates declined by 7.4% [7]. While Mozambique shares a similar neonatal mortality ratio of 27 per 1,000 live births [8] and a stillbirth rate of 17 per 1,000 total births with overall SSA [9], it has a significantly lower maternal mortality ratio (MMR) of 127 deaths per 100,000 live births compared to the overall MMR of 536 deaths per 100,000 live births in SSA [10].

Despite these improvements, maternal and neonatal mortality ratios and stillbirth rates remain unacceptably high in Mozambique with pregnancy and childbirth complications as the leading causes: 86% of maternal deaths result from direct obstetric complications [11], and 75% of newborn deaths are caused by prematurity, childbirth-related complications, and neonatal infections [12]. Most of these deaths are preventable and treatable but continue to occur at high rates in low-resource settings [13].

To address maternal and perinatal morbidity and mortality, the WHO developed the Safe Childbirth Checklist (SCC) in 2015 (see S1 Text) [13]. The SCC sets forth a structured list of evidence-based delivery practices which target the major causes of maternal deaths, neonatal deaths, and stillbirths in healthcare facilities, especially in lower- and middle-income countries (LMICs). The SCC streamlines the routine flow of childbirth delivery events into four pause points at which birth attendants ensure that they have completed essential birth practices: (a) on admission, (b) just before pushing (or just before a Caesarean-section), (c) soon after birth, and (d) just before discharge. The SCC prompts birth attendants to implement essential practices which have been shown to improve the quality of care delivered to mothers. A birth attendant’s omission of even one of the SCC items can render the mother and their newborn vulnerable to serious and potentially lethal complications.

The SCC has been implemented and evaluated in over 35 countries, demonstrating varied levels of effectiveness in reducing childbirth complications and improving maternal and newborn health outcomes [14]. Previous studies conducted in India, Ethiopia, Tanzania, Sri Lanka, Bangladesh, Kenya, Uganda, and Namibia have demonstrated that the implementation of SCC contributed to the overall improvement of the quality of care for mothers and newborns [15–21]. Key findings indicate that SCC adoption leads to increased birth attendant adherence to essential birth practices, improved inventory management for essential supplies, facilitated clinical decision-making, enhanced communication and teamwork among providers, and better management of complications. Moreover, research conducted across various settings has highlighted the significant impact of the SCC in reducing perinatal mortality and stillbirths. In Namibia, Kenya, Uganda, and Rajasthan, India, the implementation of the SCC was associated with decreased perinatal mortality, including facility-based stillbirths, very early neonatal deaths, and neonatal mortality among low-birthweight and preterm infants [18, 21, 22]. Moreover, a post-hoc analysis from the BetterBirth trial in Uttar Pradesh, India, revealed significantly lower odds of perinatal and early neonatal mortality with each additional SCC practice performed [15].

At least 11 countries in Africa have adopted and adapted the SCC: Rwanda, Ethiopia, Burkina Faso, Guinea, Côte d’Ivoire, Mali, Nigeria, Tanzania, Uganda, Kenya, and Namibia [14, 16–18, 23–25]. The experiences in these countries have provided fresh and valuable insights into local adaptations, facilitators, and barriers to successful implementation of the SCC [26, 27]. The primary facilitators of SCC implementation were characteristics inherent to the checklist itself, including its ease of completion and comprehension, and its effectiveness as a job aid for essential practices [14]. Additional enabling factors identified included leadership commitment, provider motivation, and comprehensive training and supervision regarding SCC usage [14, 23]. Barriers to SCC implementation frequently related to a shortage of clinical staff and essential birth and checklist supplies, a lack of professional training on the SCC, perceptions of increased workload due to the SCC usage, and challenges that often coincided with delivering quality maternal care [14]. Therefore, as research across multiple regions has underscored, adapting the SCC to the local context is crucial to align it with local guidelines and for its adoption by healthcare professionals. For example, in Burkina Faso and Côte d’Ivoire, health providers suggested integrating the SCC with existing tools like the partograph and displaying it in maternity wards as a reminder of critical birth practices [23]. In Kenya and Uganda, local modifications aimed at enhancing preterm birth outcomes included integrating a triage pause for initial assessments, focusing on assessing gestational age and managing preterm labor, and adjusting the SCC to better align with national care standards [25].

Despite its strong potential to improve maternal and newborn health outcomes, the SCC has not been adopted in Mozambique, one of the poorest countries in the world with major infrastructural constraints in its healthcare system, which could potentially benefit from the SCC implementation. Advancing improvements in lowering maternal and neonatal mortality, along with enhancing the overall health of the population, are key strategic aims outlined in the Mozambique Government’s 2020–2024 Five-Year Plan [4]. These objectives are also central to the UNICEF-Mozambique 2022–2026 Strategic Plan and key to the UNDP-Mozambique collaboration goals [28, 29]. Although various national guidelines specific to certain procedures and complications during childbirth exist, they are not systematically integrated as in the SCC. Moreover, little is known about current childbirth practices in Mozambique and the feasibility and acceptability of adopting the SCC in local healthcare facilities. This formative study aims to identify facilitators and barriers to potential implementation of the SCC in Mozambique, provide insights into current childbirth practices and infrastructure in the country, and guide the Mozambique Ministry of Health (MoH)’s decisions on SCC adoption and adaptation to improve MCH outcomes nationwide.

## Methods

### Study setting

In Mozambique, the public health system is organized and administered at the national, provincial, and district levels. This structure includes four levels of health facilities, each with distinct roles and capacities. Maternity care is similarly organized within this structure [30].

Primary-level health facilities, designated as health centers, serve as the primary point of contact for the population. They provide primary health care and are classified as urban or rural based on their location, with some only having the minimal capacity to perform vaginal childbirth deliveries and others not being able to do so [31]. Secondary-level hospitals, divided into district, rural, and general hospitals, provide referral care, emergency services, and surgeries. They provide more comprehensive maternity services such as assisted deliveries and basic obstetric surgeries, but their capacity to perform C-sections varies by hospital. Tertiary and quaternary-level hospitals, which include provincial, central, and referral hospitals, provide specialized care and serve as referral centers with the capacity to offer advanced and comprehensive obstetric and neonatal care, including emergency C-sections for complicated pregnancies and births.

The study was conducted in Maputo city and Manhiça district in Maputo province, Mozambique. Maputo city is the capital and the largest city of Mozambique with a population of 1.09 million in 2017 [32]. It is located at the southern end of the country, close to Mozambique’s border with Eswatini and South Africa. The city is divided into 7administrative divisions, spanning a land area of 347.69 square kilometers. Compared to the rest of the country, Maputo City is notably better equipped with health personnel and facilities. It has 37 health facilities, including 1 quaternary central hospital, 3 secondary general hospitals, and 33 primary health centers—27 urban and 6 rural [33]. Manhiça District is a rural district in Maputo Province, covering 2,373 square kilometers and located 80 kilometers north of Maputo City, with a population of two hundred thousand [34, 35]. Manhiça district has 21 primary rural health centers and health posts and 2 secondary rural, district referral hospitals [33].

Our study sites, Chamanculo General Hospital in Maputo City and Xinavane Rural Hospital in Manhiça District are both secondary hospitals offering comprehensive maternity care. While Chamanculo General Hospital does not offer C-section services, Xinavane Rural Hospital does. The maternity wards at both hospitals are divided into three areas: admission, delivery, and postpartum [31]. These areas correspond to the four pause points that the SCC uses to streamline the routine flow of childbirth delivery care: on admission, just before pushing (or C-section), soon after birth, and just before discharge. The birth attendants who participated in our study are essentially MCH nurses with midwifery skills, working 12-hour shifts [30]. They also rotate across various MCH departments within the hospitals, demonstrating proficiency in family planning, prenatal, intrapartum, and postnatal care, as well as gynecological services. Both hospitals employ a mix of different level MCH nurses, categorized by the extent of their education and training, including elementary (equals to Grade 7), basic (Grade 10), mid-level (Grade 12), and high-level (college-educated) nurses. MCH nurses with higher levels of education are equipped to manage more complex obstetric and gynecological cases, with those at the highest level being qualified to perform C-sections.

The information system in maternity care primarily consists of patient registration forms [36]. MCH nurses in maternity wards complete comprehensive registration forms for each mother, documenting clinical conditions and information from admission to discharge. These forms capture basic patient information, such as name, age, and national ID number, and clinical information, including gestational age, childbirth procedure and outcome, direct and indirect obstetric morbidity, and newborn conditions. Maternity care also incorporates data collection systems from various specific programs, such as the HIV and malaria programs [30]. From admission to the postpartum period, MCH nurses log and monitor progress of pregnancy, childbirth, postpartum conditions for mothers and newborns, and their medications.

Different guidelines are employed in different parts of the maternity ward. In general, the admission room personnel have access to guidelines for managing hypertension in pregnancy and sexually transmitted infections in pregnant women such as HIV and syphilis. The delivery room is equipped with guidelines for neonatal resuscitation. The postpartum services have guidelines for managing postpartum hypertension, postpartum infection management, and neonatal sepsis. All rooms follow guidelines for managing maternal bleeding before, during, and after childbirth. The current guidelines are specific to certain procedure or complication but are not integrated as the SCC. There is also no current standardized monitoring or reporting checklist used in the maternity wards.

The hospitals were selected as study sites for focus group discussions (FGDs) and interviews with providers taking into account the distance to the researchers’ office located in Maputo City, their capabilities to perform comprehensive maternity care, and their distinct rural and urban contexts. The inclusion of a diversity of hospitals offered a broad perspective on the varying conditions within Mozambican health facilities.

### Study design

To ensure a comprehensive perspective, this qualitative study consists of three types of participants: birth attendants, clinical administrators, and decision-makers. The study conducted four FGDs with twenty-four birth attendants and six individual interviews with clinical administrators from Xinavane Rural Hospital in Manhiça District, Maputo Province, and Chamanculo General Hospital in Maputo City, as well as eight individual interviews with decision-makers at the MoH, the Departments of Public Health for Maputo city and Maputo province, and the Association of Midwives in Mozambique. The interviews and FGDs were guided by the Consolidated Framework for Implementation Research (CFIR) and covered four of five CFIR domains: (a) individual characteristics, (b) intervention (SCC) characteristics, and the facility’s (c) outer settings and (d) inner settings [37].

### Data collection

Participants for this study were recruited using purposive sampling methods, aiming to include individuals with diverse backgrounds who were highly knowledgeable and experienced in following and implementing various policies and clinical guidelines related to childbirth practices and fulfilled the inclusion criteria and could offer valuable insights relevant to our research questions. The recruitment and data collection period took place September 16^th^, 2022 to February 10^th^, 2023. The FGDs with birth attendants and the interviews with clinical administrators were conducted at secure private offices at the two hospitals. One interview with a decision-maker was conducted via Zoom, while the other interviews with decision-makers took place either at the secure office of the Comité para a Saúde de Moçambique (Mozambique’s Health Committee) in Maputo or at the interviewees’ private offices. The clinical administrators interviewed at each clinical site included those managing MCH care. The clinical administrators also helped the study identified the birth attendants for FGDs. Each focus group comprised five to six birth attendants who met the inclusion criteria: being 18 years or older, having at least one year of experience in maternity care, availability and willingness to participate, fluency in Portuguese, and the ability and capacity to give consent. Similarly, clinical administrators and decision-makers were eligible if they had at least one year of experience managing or monitoring maternity services or MCH programs, were 18 years or older, fluent in Portuguese, available and willing to participate, and capable of giving informed consent. Decision makers were identified through the networks of our local collaborators with the Comité para Saúde de Moçambique and the Mozambique MoH. All participants were approached by a female researcher (AH, DM, or EK) and obtained written consent for participation in the interviews or the FGDs.

To assess the impact of various factors on SCC implementation, we designed the question guides of the FGD and interview based on CFIR. The questions were designed to assess current childbirth practices and infrastructure as well as the feasibility of implementing SCC to improve maternal and perinatal outcomes in Mozambique. The interview and FGD guides were tailored to the roles and responsibilities of the participants (see S2 Text). We created a pilot FGD guide and tested it to ensure that study participants could adequately contribute to a rich discussion (see S1 Table). The pilot FGD was conducted at Malhangalene Centro De Saúde (Health Center at Malhangalene) with seven birth attendants from five different health centers who did not work at the two selected clinical sites where formal data collection was to be conducted. The officers at Association of Midwives designated the birth attendants who participated in the pilot FGD. Each of them had rich prenatal-to-postnatal-care work experience from their clinical rotations in the maternity wards. We adjusted the structure and wording of the questions as needed and enhanced the moderating skills of the researchers during pilot [38].

Prior to data collection, all participants were given hard copies of the WHO SCC at least one day before the interview and FGD to familiarize themselves with its contents. After the interview and FGD, the SCC copies were collected by the researchers to avoid any unintended consequences resulting from the use of the SCC without proper instruction and support. The overall purpose of the SCC and each of its check items were explained to study participants before the FGD and interview. Participants were given opportunity before and after the FGD and interview to ask questions about the SCC and study, and those questions were subsequently addressed by the researchers. This was done to ensure all participants comprehended the content and intended use of the SCC. Participants received compensation for their participation.

Each interview and FGD lasted approximately 60 minutes, and each was scheduled at the convenience of participants, most often during their lunch breaks. All interviews and FGDs were conducted in Portuguese. A researcher (EK or DM) went through the SCC and the consent form verbatim in Portuguese before each interview or FGD and asked if there were any questions related to the study, the SCC, or the consent before the session started. Any questions raised by the participants were addressed accordingly. Participants signed written consent forms before interviews and FGDs. To assure their anonymity, participants were identified with a participant ID instead of their names during data collection and analysis. The interviews for clinical administrator and FGDs for birth attendants were conducted by two qualitative researchers, one of whom (EK) has a Doctor of Medicine degree from the School of Medicine at Eduardo Mondlane University in Mozambique and a Master of Public Health degree from Southern Medical University in China. EK had been working as a physician, district health director, and researcher at MoH since 2014, and she was also responsible for identifying and contacting the hospitals, clinical administrators, and decision-makers. The other researcher (DM) is a local research assistant has a bachelor’s degree in social science from Eduardo Mondlane University in Mozambique and is a qualitative researcher by training. The decision-maker interviews were conducted by EK and AH. AH has a Master of Public Health in Health Policy with formal qualitative study training from Yale School of Public Health in the U.S. The researchers worked in pairs during the interviews and FGDs. One served as the moderator and took detailed notes. The other researcher took comprehensive field notes and was also responsible for timekeeping. The field notes captured the behaviors and nonverbal cues of participants and, as complementary information to facilitate later data coding and analysis, described the physical spaces in which the interviews and FGDs were conducted [39].

All interviews and FGDs were recorded for later transcription, translation, and data analysis. Within 24 hours after each interview and FGD, the researchers also completed a summary report for each data collection session, including observations, personal reflections, memos, and key takeaways.

The hard copies of the research materials, such as field notes and consent forms, are stored in a locked cabinet in a locked office at Comité para a Saúde de Moçambique, and the electronic data, such as audio recordings and transcripts, were stored in Box, a secure password-protected database authorized by Yale University.

### Data analysis

The audio recordings of the interviews and FGDs were uploaded to HappyScribe, a password-protected online software, and then transcribed and translated from Portuguese to English. To ensure their accuracy and integrity, the transcriptions and translations were then carefully reviewed by a bilingual researcher, EK.

The data analysis was performed by a team of three female researchers, AH, DB, and SR, from Yale University with formal qualitative study training. The data from FGDs and clinical administrators were coded and analyzed by AH, DB, and SR, and the data from decision-makers were coded and analyzed by AH and SR. The information in transcripts that might reveal the participant’s identity was removed. The data analysis employed a rigorous deductive thematic method, enabling a thorough and nuanced analysis of the data [40]. The coding process used a deductive approach, using the pre-established CFIR codebook as a guide [37]. During the development of the codebook, exemplar quotes, enriched code definitions and descriptions, and detailed inclusion and exclusion criteria were added to the initial CFIR codebook in Microsoft Excel to provide clear guidance for the coding process and contextualize the CFIR codebook for our study.

After developing the codebook, each member of the data analysis team independently coded each transcript using the comment feature in Microsoft Word. Throughout the coding process, the data analysis team met regularly to review and discuss the coded segments line by line and resolve any discrepancies through highly participatory group discussions to achieve consensus and ensure the coding consistency. When the coding was completed in the Microsoft Word, the transcripts were imported to NVivo 14, a qualitative analysis software, and recoded to match the coding in Word. The NVivo was used to enable the retrieval of the coded segments and facilitate the systematic analysis of the codes. The data analysis team also incorporated feedback from the interviews and FGDs moderators (EK and DM) to ensure the interpretations were aligned to the data. Furthermore, the detailed narrative for each code and findings from the coding process were organized according to each of the CFIR domains. Finally, the data analysis team identified common themes across the findings categorized by the CFIR domains. These themes were then categorized into SCC implementation facilitators and barriers.

### Ethical statement

This study was approved prior to the start of data collection by the Human Subjects IRB committee at Yale University in the United States in May 2022 (IRB protocol #2000032748) and the Comité Nacional de Bioética para a Saúde in Mozambique (National Committee for Bioethics in Health, CNBS) in September 2022 (IRB protocol #00002657). Prior to collecting data, participants were provided with a consent form. EK went through the consent form in a thorough and word-for-word manner, explaining all aspects of the study, including the participants’ right to choose whether to participate, their ability to withdraw from the study at any point, the procedures in place for safeguarding the confidentiality and anonymity of their information, and the general contents of the FGD and interview. Participants were required to sign the consent forms if they wanted to participate, with one copy provided to them for their own records and another kept as part of the study documentation at the Maputo office of Comité para a Saúde de Moçambique.

### Inclusivity in global research

Additional information regarding the ethical, cultural, and scientific considerations specific to inclusivity in global research for this study is included in the Supporting Information (S1 Checklist).

### Conceptual framework

The CFIR examines the implementation environment of an intervention, and how to facilitate its effective implementation through the lens of five domains, (a) intervention characteristics, (b) outer setting, (c) inner setting, (d) individuals’ characteristics, and (e) implementation process [37]. As this is a formative study to assess the feasibility of SCC implementation, we excluded the implementation process domain as the SCC has not yet been implemented. Among the four domains, we identified eleven constructs that are relevant to our study for analyzing the qualitative data: (a) intervention characteristics (complexity, adaptability, relative advantage, and innovation cost), (b) outer setting (policies and laws, partnerships and connections, and societal pressure), (c) inner setting (compatibility, available resources, and culture), and (d) characteristics of individuals (knowledge and beliefs about the intervention).

## Results

Twenty-four birth attendants participated in the FGDs, and six clinical administrators and eight decision-makers took part in the individual interviews. As no new information emerged after the four FGDs and fourteen individual interviews, we considered that information saturation was reached. The duration of FGDs ranged from 39 minutes to 62 minutes, and interview time ranged from 26 minutes to 70 minutes. Detailed sociodemographic characteristics of the participants are presented in Table 1.

**Table 1:** Characteristics of participants in Maputo City and Maputo Province, Mozambique.

***1*.*1 Birth Attendants (n = 24)***	
*Xinavane Rural Hospital (n = 12)*	
Gender (%)	
Female	100
Age, mean (SD)	37.1 (7.6)
Years of experience working in maternity wards, mean (SD)	4.1 (4.1)
Education level (No. of participants)	
Elementary level (Grade 7) of nursing school	2
Basic level (Grade 10) of nursing school	0
Middle level (Grade 12) of nursing school	9
High level (College-level education) of nursing school	1
*Chamanculo General Hospital (n = 12)*	
Gender (%)	
Female	100
Age, mean (SD)	36.4 (6.4)
Years of experience working in maternity wards, mean (SD)	7.8 (4.2)
Education level (No. of participants)	
Elementary level (Grade 7) of nursing school	0
Basic level (Grade 10) of nursing school	1
Middle level (Grade 12) of nursing school	10
High level (College-level education) of nursing school	1
***1*.*2 Clinical Administrators (n = 6)***	
Gender (%)	
Female	100
Age, mean (SD)	38.2 (11.8)
Years of experience working in maternity wards, mean (SD)	13.6 (12.9)
Education level (No. of participants)	
Bachelor’s degree	4
Doctor of Medicine	2
***1*.*3 Decision-makers (n = 8)***	
Gender (No. of participants)	
Female	7
Male	1
Age, mean (SD)	38.9 (8.1)
Years of experience working in maternity wards, mean (SD)	13 (9.2)
Education level (No. of participants)	
Bachelor’s degree	3
Doctor of Medicine	5

All codes identified from the transcripts were mapped to CFIR constructs. Of the 48 CFIR constructs assessed, eleven were determined to be relevant barriers and/or facilitators to implementing the SCC. Specifically, one CFIR construct addressed facilitators (complexity), and five CFIR constructs addressed barriers (adaptability, relative advantage, innovation cost, available resources, and societal pressure). Six other CFIR constructs addressed both facilitators and barriers (policies and laws, partnerships and connections, compatibility, culture, and knowledge and beliefs about the intervention). The study findings were organized by themes below, and Table 2 linked the barriers and facilitators of the SCC implementation to specific CFIR constructs.

**Table 2:** Barriers and facilitators of implementing the WHO Safe Childbirth Checklist (SCC) in Maputo City and Maputo Province, Mozambique.

CFIR Domain/Constructs	Barrier & Facilitators	
**Intervention Characteristics**		
Complexity	Facilitator	The SCC is simple and easy to understand
Adaptability	Barrier	The SCC needs to be contextualized to better align with the specific needs and context
Relative Advantage	Barrier	Participants found the SCC redundant with their current work routine
Innovation Cost	Barrier	The SCC implementation requires additional costs
**Outer Setting**		
Policies and Laws	Facilitator	The SCC objectives aligned with MoH guidelines
	Facilitator	Regular supervision and technical support visits from MoH may help monitor and improve the maternity services quality
	Barrier	Inequitable regional resources distribution may limit resources for the SCC adoption
Partnerships and Connections	Facilitator	Local community and leadership can disseminate information on SCC and motivate providers
	Barrier	Inadequate and inequitable support from external funders and partners
Societal Pressure	Barrier	Misunderstanding from patients and low recognition in society may lower the motivation for the SCC implementation
**Inner Setting**		
Compatibility	Facilitator	The childbirth concepts in the SCC are consistent with those of the maternity unit
	Barrier	The SCC may add to the already heavy paperwork
	Barrier	The SCC may not fit with the existing workflows in the maternity ward
Available Resources	Barrier	A severe shortage of human and material resources may hinder SCC implementation
Culture	Facilitator	Regular internal committee meetings may improve maternity services quality
	Facilitator	Participants were dedicated to improving childbirth practices and open to SCC implementation
	Barrier	Participants resisted accepting the new changes from the SCC, finding them intimidating
**Characteristics of Individual**		
Knowledge & Beliefs	Facilitator	Participants were familiar with the knowledge and skills required by the SCC
	Facilitator	Participants believed SCC would improve the quality of childbirth practices
	Barrier	Participants were concerned the SCC would increase the workload
	Barrier	Participants worried about penalties for not completing the SCC

### Facilitators

#### The SCC is simple and easy to understand

When participants were asked about the complexity of the SCC, they agreed that the content and format of the checklist were easy to understand.

*“I don’t think it is complicated at all. It just has basic aspects of everyday life in a maternity ward, or the day-to-day life of a midwife, or a nurse, so I don’t think it’s complicated. It’s direct, it has very concrete aspects.”*

(Decision-maker 5)

#### The SCC aligned with the national maternal and child health agenda

The participants stated that the current MoH guidelines and efforts were consistent and reflected in the SCC objectives, indicating that the SCC implementation aligned with the national maternal and child health agenda.

*“In general, one (SCC) is applying what are practices according to the MoH guideline, which is humanized childbirth or humanization of childbirth… All nurses have this orientation.”*

(Clinical Administrator 5)

Participants mentioned that strong support and commitment from both the local community and public health leaders about safe childbirth were crucial, as they can significantly contribute to spreading information on MCH and motivate clinics and birth attendants to engage in the SCC implementation effort.

*“We, in the community, have the community leaders, maternal health nursing component, and the traditional midwives. They help information dissemination of the maternal and child health package… We will be able to involve them, to know that there is a checklist… so that they can help the dissemination of information.”*

(Decision-maker 4)

Furthermore, decision-makers emphasized that the MoH undertook regular supervision visits, offered technical support, and conducted in-service training at clinics. These initiatives are designed to ensure guidelines compliance and improve service quality in maternity wards. Such efforts aligned with the objectives of the SCC and may aid in its effective implementation.

*“We do the monitoring of the activities, supervision… both scheduled supervisions and surprise visits. We make surprise visits to maternity hospitals, mainly to check if in fact they are doing their job well… We also reinforce it with some in-service training. When we get there, in these supervisions, we also explain: ‘Look, you are not doing it right here.’ We correct what is good practice and follow up on the needs.”*

(Decision-maker 1)

#### Participants had positive beliefs about the SCC

During the interviews and FGDs, the participants displayed a strong understanding, wealth of knowledge, and a high level of professionalism and dedication to improving the quality of childbirth practices. They were open to updating their knowledge using the SCC and acknowledged the importance of continuously learning and keeping themselves informed.

*“Science is dynamic. There are things that are being abolished and things that are being introduced. So, I try to say you should implement this study, while one thing or another could be abolished, so as we are here the council, we are here today to learn…. Let’s give progress to this study.”*

(Birth Attendant 8)

Moreover, participants expressed confidence that the implementation of SCC would lead to positive changes in current practices and result in improved quality of maternity services.

*“I think that the list has a format that goes according to what we are talking about, because what we need is a standard procedure for the teams. Then, for the complications, we will have more trained people, but we also need team with a minimum standard procedure, and the list is simple. It is a list that reduces the time of work or procedure of the colleague… I find the list simple and sufficient.”*

(Decision-maker 7)

### Barriers

#### The SCC was viewed as redundant

Participants expressed that the SCC did not offer a significant advantage over their current work routine, viewing it as an additional form to fill out and adding to the workload of birth attendants.

*“It would be one more instrument. It would be a repetition of what we already do… All these flowcharts that we have already exist. And that is exactly what we do. And it looks like we don’t read it, because this, because that, but no. We already do that … We end up having less time to do our activities, to exercise the technique. We stay longer, we have [to] read and write, which doesn’t help us much either. It is very tiring.”*

(Birth Attendant 3)

“I don’t think that the Checklist, by itself, will meet the needs… what will happen is this list will be one more paper in the maternity ward… The form alone is not going to change anything. It is one more piece of paper, it is going to be one more tool. As I said, the current guideline already recommends many of these questions, and they are in the form. What do we do? We fill it out, fill it out, fill it out.”

(Decision-maker 6)

#### The SCC might be incompatible with the current workflows

The concepts and practices outlined in the SCC were found to be mostly consistent with current practices in the maternity unit according to the participants. Filling out the SCC itself, however, is likely to impede existing workflows due to human resource shortages and time constraints. Participants expressed concerns about how to allocate time for other clinical activities and fill out the SCC, as there may be competing priorities.

*“Because of the overload of work, one or another thing ends up slipping away… We have gynecology, maternity, c-sections, pathological pregnancy, gynecology, admission, delivery room, it’s for one nurse… So, everything that happens there ends up exhausting your knowledge, and your strength, you don’t know what to do…. It’s not because she is unwelcome [the SCC], she is welcome, yes. But treating the person himself, the work, it becomes difficult to follow the form.”*

(Birth Attendant 9)

Moreover, participants expressed concern about the workload related to paperwork. They already had a significant amount of paperwork to fill out, and the addition of SCC might increase their workload. Some participants suggested simplifying the current paperwork instead of introducing a new one.

*“It is complicated because we already have many instruments. If the list doesn’t come to remove anything, it comes to add, it’s another job… Now, if the list comes and reduces the work for us, and summarizes a lot of things, it is welcome. If it is to add to it, it will not make us comfortable.”*

(Clinical Administrator 4)

#### The SCC needs to be better aligned with the context

Although the SCC was viewed as simple and easy to understand, participants voiced the need to adapt it to the local context. Participants proposed multiple adaptations to integrate the SCC into their work routines and contexts, enhancing its implementation feasibility. These adaptations included transforming the SCC into a pocketbook rather than adding it to existing paperwork, displaying it as a wall poster, incorporating a section explaining incomplete practices, using it to evaluate supply availability, and merging it with existing tools like the patient clinical registration form, which includes medical history and diagnoses.

*“My suggestion would be that it should be in a format like these HIV flowcharts, for example. You don’t make us waste time even opening a document and looking for how to do it. Then nail it to the wall…the person looks, sees the explanations and does it. It is easier to do than in the form of a list.”*

(Clinical Administrator 2)

“Or maybe one could think of a decentralized instrument, which could perhaps feed into another instrument already at the central level… If we had an instrument that helps us to check what is the quality of the work of our maternity ward… And maybe to send the information to the central level as well, to see what is happening, what is failing, which is to take the proper precautions.”

(Clinical Administrator 5)

#### Inadequate external support may hinder SCC implementation

Participants emphasized that the external financial support from the MoH to maternal health care was inadequate, and the assistance from funders and partners was distributed unevenly across the country, often focused on specific diseases in a vertical manner. This could impede the adoption and implementation of the SCC, given that maternal health care is currently underfunded and not given priority.

*“The financial allocation for the reduction of maternal and child mortality in a direct way is minimal, is reduced, and is ineffective. We have a maternal and child health plan in a year that cannot meet 50% of the needs… The use of external funds, which is far from the Paris Declaration, we don’t have much flexibility of funds to decide where they are allocated. The care area is underfunded, and it is the area that we should improve. We have pillars that are necessary [to be improved, including] educating how the delivery has to be, pregnancy care, the significance of various stages of pregnancy, labor expectations, pain management, and practices."*

(Decision-maker 7)

With the specific pillars the decision-maker highlighted also being key elements in the SCC, the current lack of financial support for maternal and child health care could signal potential challenges in implementing the SCC.

#### Resource shortfalls may impede SCC implementation Resource shortfalls may impede SCC implementation

A major barrier to the SCC implementation is the limited availability of resources, including human resources, materials, physical space, and professional training. Despite the perceived benefits of SCC, the severe shortage of resources makes it challenging to successfully implement the SCC in clinical settings.

*“What we need in Mozambique, in fact, is more equipped rooms, more spacious rooms, because our infrastructure sometimes does not create these types of conditions for a well-designed guideline. The strategies are well designed, but our conditions don’t help us, they don’t favor us having this model birth (SCC) that we are talking about, which would be better.”*

(Decision-maker 3)

Notably, participants expressed concern that the implementation of SCC would further increase their overwhelming workload as there is typically only one nurse per shift in the maternity unit, responsible for caring for both mothers and newborns. The already serious staff shortage could not accommodate the addition of another instrument that might increase the provider burnout. Allocating scarce time to complete the SCC would further increase staff workload.

*“The implementation of the list is not bad. But as we were just saying… the lack of human resources, I think that this list will be more of an overload, an extra work, where the staff at that moment are few… But the list is not bad. It is very good, it helps. It is the moment when someone can forget something, looking here, sees that here is something that can be done or should be done. But looking at the work you already have in the maternity ward, it’s a lot. There are many documents to be filled out. One more document, it’s more overload.”*

(Birth Attendant 18)

*“We would feel overwhelmed. [The nurse] couldn’t fill out… and she is going to be overload. How is it? She will even ask herself, ‘but can’t you see? Because I am all alone.’”*

(Birth Attendant 17)

The scarcity of essential birth supplies in the maternity ward posed another significant barrier to implementing the SCC and achieve its purpose to enhance the quality of childbirth practices.

*“For the maternity case, we are missing too many antihypertensives. Just talk about methyldopa, hydralazine, dihydralazine… and this has made our work very difficult.”*

(Clinical Administrator 5)

*“There are no gloves. How will it go well? How will you take care of yourself? How will you comply with what the document [SCC] asks for?”*

(Clinical Administrator 1)

Meanwhile, the participants highlighted the importance of professional training for successful SCC implementation and requested refresher training to improve their knowledge and skills.

*“I think that if the people who are [going] to use the checklist are not very well trained, they can have a complication because it [the SCC] can be filled out not in the same standard way. The training of the people who are going to use the form itself needs to be standardized.”*

(Clinical Administrator 4)

Furthermore, the cost of the SCC implementation poses another challenge. The health facilities in Mozambique have very limited resources, and the costs of reproducing, distributing, storing, and completing the SCC, including expenses such as printers, paper, and storage space, could add an additional financial burden on the clinics.

*“The list is produced, and then it is the health unit’s responsibility to reproduce it. And that doesn’t go very far, because we will see that the health unit doesn’t have the capacity to reproduce the form itself…It is already difficult for the health unit to continue because they are not all able to multiply their own records.”*

(Clinical Administrator 4)

#### Low motivation and societal pressures deter providers from adopting SCC

Participants indicated that their existing workload, particularly with paperwork and completing instruments, was already overwhelming. They expressed concerns about their ability to properly fill out additional forms, suggesting that introducing a new instrument could be daunting.

*“We get blinded in front of a document. Many times, we get scared just by looking at the document. Do this, we have to fill it out like this. Sometimes we fill it out, but not properly as it should be.”*

(Birth Attendant 10)

*“Whenever we get a new instrument, there is resistance in change, because at some point, the nurses have to give their reasons because they have too many instruments to be able to fill out, to be able to check. When more than one instrument arrives, they get a little tired, a little angry, because we have many books to fill in.”*

(Decision-maker1)

Additionally, some participants expressed concerns that failure to fill out the SCC could result in penalties or other negative consequences.

*“It would be possible [to implement the SCC]. It would help some, but it could also penalize us for things that are not our level of competence to resolve, such as the issue of lack of medicines, lack of running water, at some point in the anesthesia machine, a shortage of operating room staff.”*

(Clinical Administrator 5)

Moreover, many birth attendants reported experiencing stigma and pressure from mass media, local community, and patients, which further limited their motivation to adopt another instrument like SCC and improve the quality of the maternal and child health services.

The participants expressed that the social recognition of birth attendants was low, and this lack of recognition was a demoralizing factor in their work. Despite the birth attendants’ strong desire to improve their work and adopt SCC, they felt that their efforts were not valued or recognized by the community.

*“Because if we look at the media, they are against us. Just for someone to be born outside, we are already on television. But if I attend childbirth outside without gloves to help, I won’t be on television. But if someone is born outside, even five meters from the hospital, we are going to be smeared with all of this. ‘Chamanculo is negligent, there was no emergency room.’ So, motivation factor.”*

(Birth Attendant 15)

There were instances in which the companions or patients complained the practices of the birth attendants, resulting in the spreading of negative comments about the birth attendants in the community, further diminishing their motivation to work.

*“Even being a woman, a companion [of the delivery mother] doesn’t understand what happens inside the maternity ward. Even the techniques that the nurse will perform, she thinks you’re mistreating that person… She starts talking bad about us in the community.”*

(Birth Attendant 6)

## Discussion

### Main findings and interpretation

This formative qualitative study sought to identify potential facilitators and barriers to implementing the SCC in the context of the childbirth practices and conditions in Mozambique at the time this study was conducted. The study explored the feasibility of SCC implementation by assessing the initial knowledge and attitudes of a diverse group of stakeholders from various professional backgrounds.

The barriers and facilitators identified in our study agree with most of the findings from the countries where the SCC had been tested before [14, 21, 26, 27, 41]. The common facilitators of SCC use were related to the checklist itself, as it’s easy to complete and acts as a useful reminder for essential childbirth practices that aligned with the national and local guidelines [14]. The major barriers were linked to local challenges, including insufficient material and human resources, inadequate training, perceptions of increased workload associated with the SCC use, lack of staff motivation to use SCC, and an underfunded MCH care [14, 21, 27, 41, 42].

In Mozambique, due primarily to the structural challenges of the overall health system, the implementation of SCC faces multiple obstacles. Support for MCH care from the MoH and external funders was found to be inadequate and not given priority, with resource distribution often focused on specific diseases through a vertical approach. This lack of funding for maternal care might further limit the resources available for adopting SCC and hindered the implementation of quality, evidence-based delivery practices required by SCC. Clinics in our study commonly faced shortages of essential medicines, equipment, and materials needed for critical childbirth practices. Additionally, the costs associated with reproducing, distributing, storing, and completing the SCC imposed an extra financial burden on the already under-resourced maternity services in the clinics. Moreover, given that there was often only one birth attendant per shift in the maternity ward, implementing and completing the SCC may have competed with other clinical activities for the limited time, resources, and attention of the birth attendant. As a result, birth attendants viewed the SCC as redundant, feeling it added to their workload without offering significant advantages over their current practices. They also found the prospect of introducing another instrument daunting, given the already substantial paperwork in the clinics. Additionally, there was concern that failing to complete the SCC could lead to penalties.

Meanwhile, mothers’ mistrust and perceived poor quality of care have led to blame directed at birth attendants, which may have contributed to their low motivation. Negative comments from the community further undermine the birth attendants’ social recognition and increase societal pressure on them. Our study participants highlighted poor morale, weak motivation, and low recognition among the primary reasons for their reluctance to adopt another protocol like the SCC, in the context of their already overwhelming workload. These barriers need to be addressed to facilitate the SCC implementation in Mozambique.

**Fig 1:**
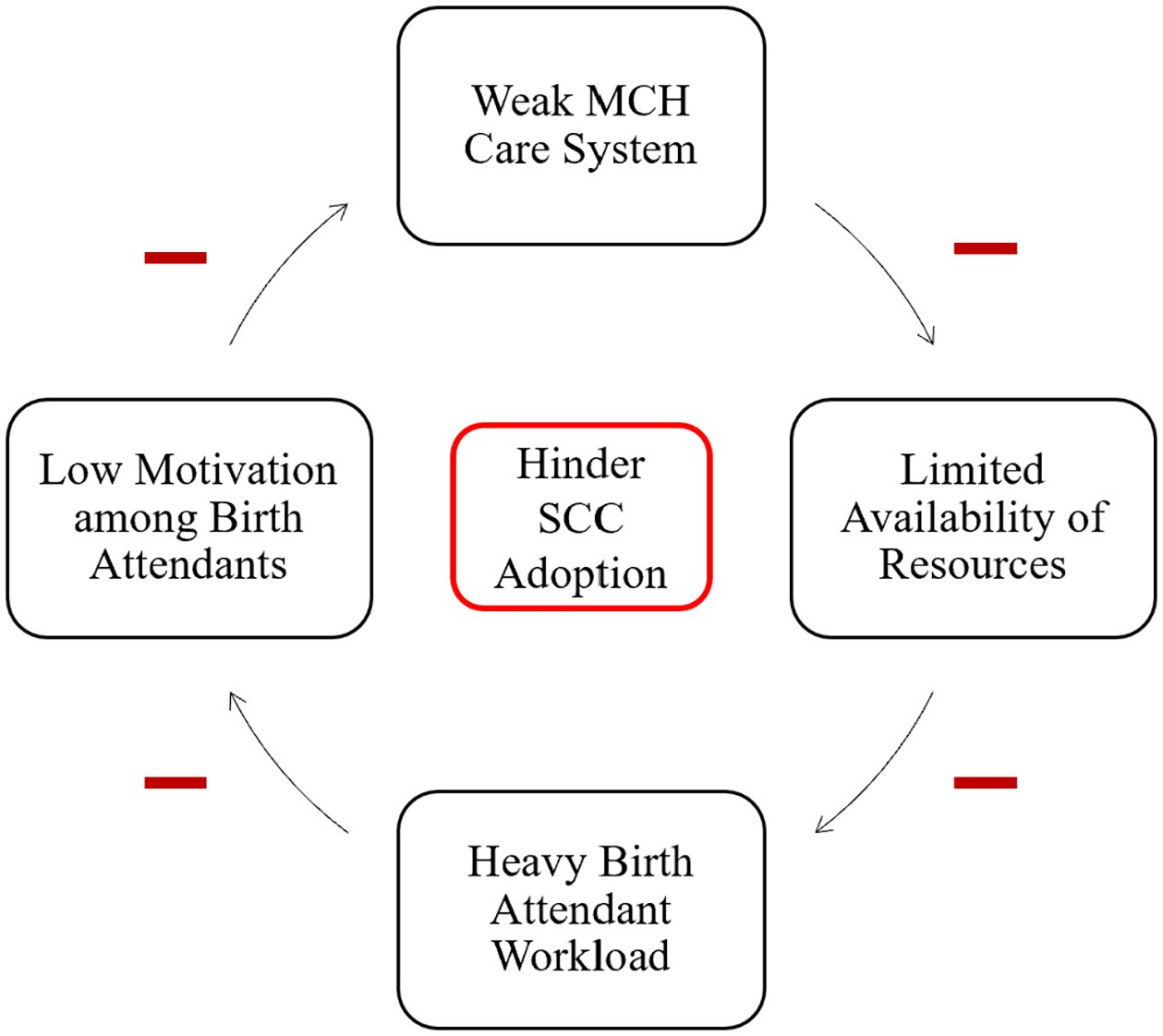
Negative feedback loops hindering the adoption and the implementation of the SCC.

We recognized that implementing the SCC in our study context involves many interacting factors that potentially reinforce each other within a dynamic system. Therefore, we hypothesized that there were negative feedback loops that hindered the health system’s ability to implement the SCC. Informed by our findings we further hypothesize that these feedback loops were likely to be (a) a weak MCH care system, (b) limited availability of resources, (c) heavy birth attendant workload, and (d) low motivation among birth attendants (Fig 1). Our hypotheses are consistent with findings from a previous study conducted in Nampula Mozambique seeking to understand how to improve breast feeding counseling through the health system [43], highlighting the fact that our findings have implications beyond just the SCC.

Despite these obstacles, many birth attendants remained committed to improving the quality of childbirth practices and adopting SCC. They recognized that the SCC aligned with the national MCH goals and the need to continue educating themselves. Birth attendants did not express the need for pay-for-performance for filling out SCC but suggested that allocating more financial resources towards creating better working conditions and strengthening the healthcare system would be helpful. Moreover, participants suggested modifying the format of SCC, such as displaying it as a poster in the maternity ward or integrating it into existing tools like the patient clinical registration form. This would help contextualize SCC’s use, better integrate it into the health providers work routines and facilitate its implementation. However, it is possible that altering the use and format of the SCC might contribute to potential changes in its original purpose and affect its efficacy.

#### Limitations and strengths

This study has several limitations. Firstly, since participants lacked real-life experience in SCC implementation, the barriers and facilitators identified were not directly informed by their experience of using the SCC. Moreover, without implementing the SCC, this formative research study was not able to assess the actual implementation process domain of the CFIR or identify potential effective activities utilized in the SCC implementation [37]. However, we provided the SCC to participants at least one day before the interviews and FGDs and explained the purposes of the SCC iteratively before and throughout the interviews and FGDs to facilitate their understanding of its content and use. While we could not confirm whether participants had read the SCC beforehand, we took steps to ensure their understanding of its purpose and checklist items. Before each FGD and interview, participants were explained the purpose of the SCC and each of its checklist items. Researchers addressed the participants’ questions to ensure that they all understood the content and intended use of the SCC before proceeding with, and during and after the interviews and FGDs were conducted.

Moreover, it’s important to highlight that in our study participants were deliberately chosen for their extensive knowledge and experience in adhering to and implementing various clinical guidelines related to childbirth practices and policies. As frontline health workers and policymakers, they had extensive familiarity with the objective and integrated content of the SCC. During the FGDs and interviews, they indeed indicated that although the SCC might present a new format as a clinical checklist, the content was familiar to them. Additionally, based on their experience they were able to identify specific items in the SCC that they felt would be challenging and provided substantive feedback on these items during the discussions.

Secondly, we sampled one rural and one urban hospital in Maputo Province and Maputo City, aiming to represent varying conditions in health facilities. Nonetheless, our sample may not fully capture the reality across Mozambique, given the substantial differences in health care quality and access across the country, the external validity of our findings must be interpreted with caution. Moving forward, future SCC studies in Mozambique should include various levels and types of health facilities, including primary health centers, from different regions of the country.

Thirdly, while we hypothesized the presence of several negative feedback loops involving barriers from system-level to individual-level that may make SCC implementation challenging, we acknowledge that this hypothesis needs to be confirmed through further research as causal relationships cannot be established through a qualitative study. We further recognize that the hypothesized feedback loops are an oversimplified representation of barriers to SCC implementation. Further research will also be needed to understand how to counteract negative with positive feedback loops to enable SCC implementation in the context of under-resourced maternity healthcare systems.

Lastly, we fully acknowledge that it will be crucial to include the views of women and the community in the co-design of the SCC implementation process in Mozambique. As an initial formative study, we chose to concentrate first on the perspectives of birth attendants, clinical administrators, and decision-makers in Mozambique, aligning with the clinical context where the SCC is intended to be applied. Future community-engaged co-design studies conducted by our team will incorporate the voices of local women and the community to ensure comprehensive and inclusive insights.

Despite the limitations, this study has several strengths. While our findings confirm findings previously reported in other countries, this study stands out as the sole formative qualitative study that was conducted prior to actual SCC implementation and the first SCC study conducted in Mozambique. Our approach aligns closely with the WHO Safe Childbirth Checklist Implementation Guide [44], emphasizing the necessity of assessing available resources and current practices prior to large-scale implementation to determine how the SCC can be optimally employed and what prerequisites must be met for its success.

Conducting this study before SCC implementation offers several benefits. This formative study reflects a commitment to ensuring that SCC implementation aligns with and addresses the country’s specific needs. As reported by a previous study, SCC implementation might increase the workload and frustration of birth attendants [21]. Ignoring this clear finding confirmed in our study could inadvertently generate unintended consequences within local communities and the MCH care system in Mozambique.

Moreover, our study was carried out in close collaboration with Mozambique’s MoH based on the principles of mutual respect and benefit, equitable communication, and productive dialogue between the global health research team and the local partners, with a commitment to reporting our findings to local healthcare leadership [45]. The findings of this study have been presented to the decision-makers and researchers in Mozambique and will be further disseminated in the country to assist the MoH in determining the next steps for SCC implementation. We expect for our findings to support a co-design phase of an initiative to implement the SCC in Mozambique.

#### Implications

Our study identified severe health care systems resource shortage as a key barrier to the SCC implementation in Mozambique, emphasizing the need to reconsider the focus of MCH studies and research methods used. Unlike the typical practice of conducting pre-post-implementation studies or randomized controlled trials (RCTs) to investigate facilitators and barriers for SCC implementation, our study shows that a proactive pre-implementation assessment can provide equally important contextual insights. Furthermore, conducting pre-implementation assessments could inform resource allocation strategies to address critical gaps in human and material resources for the SCC implementation with the ultimate goal of strengthening the overall MCH care system.

Furthermore, given that numerous barriers to SCC implementation are fundamentally linked to the shortcomings of Mozambique’s healthcare system, we call for future funders and partners shifting their focus from vertical funding to initiatives that prioritize the provision of essential materials, human resources, and professional training in primary care. Moreover, recognizing that there is no one-size-fits-all model for SCC implementation due to various contexts, future implementation research should include different types of health facilities and various levels of healthcare systems across Mozambique. Future research should take into account what we have learned from our study in Maputo City and Maputo Province and determine the optimal complementary intervention packages to adapt SCC implementation strategies to the country’s unique settings [42], taking the voices of women and communities fully into account.

In conclusion, our innovative study has played a crucial role in empowering local providers by listening to their voices and engaging them in the decision-making process for the implementation of the SCC in Mozambique. Their contributions have highlighted the urgent need for improving the quality of MCH care and enhancing the capacity of the health system in the country. Moreover, our study has identified various key factors that are vital for the successful implementation of the SCC, which include ensuring the availability of adequate human and material resources, providing comprehensive professional training, adapting the SCC contextually, maintaining strong political commitment, and garnering support from equitable partnerships. Lastly, we call for future research to undertake a holistic evaluation of the local context prior to the implementation of the SCC, thereby promoting decolonized global health research and practice and ensuring that interventions are contextually relevant and culturally sensitive.

## Supporting information

S1 Text WHO Safe Childbirth Checklist. (PDF)

S2 Text Question guides for FGDs and interviews. (DOCX)

S1 Table Codebook and question guide for pilot FGD. (XLSX)

S1 Checklist PLOS questionnaire on inclusivity in global research. (DOCX)
