# Supplementary material for: Barriers and facilitators for implementing the WHO Safe Childbirth Checklist (SCC) in Mozambique: A qualitative study using the Consolidated Framework for Implementation Research (CFIR)
Source: PLOS Glob Public Health. 2024 Sep 5;4(9):e0003174. doi: 10.1371/journal.pgph.0003174 (PMC11376584; doi:10.1371/journal.pgph.0003174)
Supplement: S1 Text — (PDF) [file pgph.0003174.s001.pdf]

BEFORE BIRTH

WHO Safe Childbirth Checklist

World Health Organization

1 On Admission

**Does mother need referral?**<br>☐ No<br>☐ Yes, organized	Check your facility's criteria
**Partograph started?**<br>☐ No, will start when ≥4cm<br>☐ Yes	Start plotting when cervix ≥4 cm, then cervix should dilate ≥1 cm/hr<br>• Every 30 min: plot HR, contractions, fetal HR<br>• Every 2 hrs: plot temperature<br>• Every 4 hrs: plot BP
**Does mother need to start:**<br>*Antibiotics?*<br>☐ No<br>☐ Yes, given	Ask for allergies before administration of any medication<br>Give antibiotics to mother if any of:<br>• Mother's temperature ≥38°C<br>• History of foul-smelling vaginal discharge<br>• Rupture of membranes >18 hrs
Magnesium sulfate and antihypertensive treatment?<br>☐ No<br>☐ Yes, magnesium sulfate given<br>☐ Yes, antihypertensive medication given	Give magnesium sulfate to mother if any of:<br>• Diastolic BP ≥110 mmHg and 3+ proteinuria<br>• Diastolic BP ≥90 mmHg, 2+ proteinuria, and any: severe headache, visual disturbance, epigastric pain<br><br>Give antihypertensive medication to mother if systolic BP >160 mmHg<br>• Goal: keep BP <150/100 mmHg
☐ **Confirm supplies are available to clean hands and wear gloves for each vaginal exam.**	
☐ **Encourage birth companion to be present at birth.**	
☐ **Confirm that mother or companion will call for help during labour if needed.**	Call for help if any of:<br>• Bleeding<br>• Severe abdominal pain<br>• Severe headache or visual disturbance<br>• Unable to urinate<br>• Urge to push

This checklist is not intended to be comprehensive and should not replace the case notes or partograph. Additions and modifications to fit local practice are encouraged. For more information on recommended use of the checklist, please refer to the "WHO Safe Childbirth Checklist Implementation Guide" at: www.who.int/patientsafety.

© WHO 2015
WHO/HIS/SDS/2015.26

Completed by ____________________

WHO Safe Childbirth Checklist

World Health Organization

2 Just Before Pushing (Or Before Caesarean)

Does mother need to start:

Antibiotics?

☐ No
☐ Yes, given

Ask for allergies before administration of any medication
Give antibiotics to mother if any of:
- Mother's temperature ≥38 °C
- History of foul-smelling vaginal discharge
- Rupture of membranes >18 hrs
- Caesarean section

Magnesium sulfate and antihypertensive treatment?

☐ No
☐ Yes, magnesium sulfate given
☐ Yes, antihypertensive medication given

Give magnesium sulfate to mother if any of:
- Diastolic BP ≥110 mmHg and 3+ proteinuria
- Diastolic BP ≥90 mmHg, 2+ proteinuria, and any: severe headache, visual disturbance, epigastric pain

Give antihypertensive medication to mother if systolic BP >160 mmHg
- Goal: keep BP <150/100 mmHg

Confirm essential supplies are at bedside and prepare for delivery:

For mother

☐ Gloves
☐ Alcohol-based handrub or soap and clean water
☐ Oxytocin 10 units in syringe

Prepare to care for mother immediately after birth:
Confirm single baby only (not multiple birth)
1. Give oxytocin within 1 minute after birth
2. Deliver placenta 1-3 minutes after birth
3. Massage uterus after placenta is delivered
4. Confirm uterus is contracted

For baby

☐ Clean towel
☐ Tie or cord clamp
☐ Sterile blade to cut cord
☐ Suction device
☐ Bag-and-mask

Prepare to care for baby immediately after birth:
1. Dry baby, keep warm
2. If not breathing, stimulate and clear airway
3. If still not breathing:
   - clamp and cut cord
   - clean airway if necessary
   - ventilate with bag-and-mask
   - shout for help

☐ **Assistant identified and ready to help at birth if needed.**

This checklist is not intended to be comprehensive and should not replace the case notes or partograph. Additions and modifications to fit local practice are encouraged. For more information on recommended use of the checklist, please refer to the "WHO Safe Childbirth Checklist Implementation Guide" at: www.who.int/patientsafety.

Completed by ______________________

WHO Safe Childbirth Checklist

3 Soon After Birth (Within 1 Hour)

**Is mother bleeding abnormally?**<br>☐ No<br>☐ Yes, shout for help	If bleeding abnormally:<br>• Massage uterus<br>• Consider more uterotonic<br>• Start IV fluids and keep mother warm<br>• Treat cause: uterine atony, retained placenta/fragments, vaginal tear, uterine rupture
**Does mother need to start:**<br>*Antibiotics?*<br>☐ No<br>☐ Yes, given	Ask for allergies before administration of any medication<br>Give antibiotics to mother if placenta manually removed or if mother's temperature ≥38 °C and any of:<br>• Chills<br>• Foul-smelling vaginal discharge<br><br>If the mother has a third or fourth degree of perineal tear give antibiotics to prevent infection
Magnesium sulfate and antihypertensive treatment?<br>☐ No<br>☐ Yes, magnesium sulfate given<br>☐ Yes, antihypertensive medication given	Give magnesium sulfate to mother if any of:<br>• Diastolic BP ≥110 mmHg and 3+ proteinuria<br>• Diastolic BP ≥90 mmHg, 2+ proteinuria, and any: severe headache, visual disturbance, epigastric pain<br><br>Give antihypertensive medication to mother if systolic BP >160 mmHg<br>• Goal: keep BP <150/100 mmHg
**Does baby need:**<br>*Referral?*<br>☐ No<br>☐ Yes, organized	Check your facility's criteria.
Antibiotics?<br>☐ No<br>☐ Yes, given	Give baby antibiotics if antibiotics given to mother for treatment of maternal infection during childbirth or if baby has any of:<br>• Respiratory rate >60/min or <30/min<br>• Chest in-drawing, grunting, or convulsions<br>• Poor movement on stimulation<br>• Baby's temperature <35 °C (and not rising after warming) or baby's temperature ≥38 °C
Special care and monitoring?<br>☐ No<br>☐ Yes, organized	Arrange special care/monitoring for baby if any:<br>• More than 1 month early<br>• Birth weight <2500 grams<br>• Needs antibiotics<br>• Required resuscitation

☐ **Started breastfeeding and skin-to-skin contact (if mother and baby are well).**

☐ **Confirm mother / companion will call for help if danger signs present.**

Responsibility for the interpretation and use of the material in this checklist lies with the reader. In no event shall the World Health Organization be liable for damages arising from its use. For more information visit www.who.int/patientsafety.

Completed by ______________________

AFTER BIRTH

WHO Safe Childbirth Checklist

World Health Organization

4 Before Discharge

☐ **Confirm stay at facility for 24 hours after delivery.**

Does mother need to start antibiotics?

☐ No
☐ Yes, given and delay discharge

Ask for allergies before administration of any medication
Give antibiotics to mother if any of:
- Mother's temperature ≥38 °C
- Foul-smelling vaginal discharge

Is mother's blood pressure normal?

☐ No, treat and delay discharge
☐ Yes

Give magnesium sulfate to mother if any of:
- Diastolic BP ≥110 mmHg and 3+ proteinuria
- Diastolic BP ≥90 mmHg, 2+ proteinuria, and any: severe headache, visual disturbance, epigastric pain

Give antihypertensive medication to mother if systolic BP >160 mmHg
- Goal: keep BP <150/100 mmHg

Is mother bleeding abnormally?

☐ No
☐ Yes, treat and delay discharge

If pulse >110 beats per minute and blood pressure <90 mmHg
- Start IV and keep mother warm
- Treat cause (hypovolemic shock)

Does baby need to start antibiotics?

☐ No
☐ Yes, give antibiotics, delay discharge, give special care

Give antibiotics to baby if any of:
- Respiratory rate >60/min or <30/min
- Chest in-drawing, grunting, or convulsions
- Poor movement on stimulation
- Baby's temperature <35°C (and not rising after warming) or baby's temperature ≥38°C
- Stopped breastfeeding well
- Umbilicus redness extending to skin or draining pus

Is baby feeding well?

☐ No, establish good breastfeeding practices and delay discharge
☐ Yes

☐ **Discuss and offer family planning options to mother.**

☐ **Arrange follow-up and confirm mother / companion will seek help if danger signs appear after discharge.**

Danger Signs

Mother *has any of:*
- Bleeding
- Severe abdominal pain
- Severe headache or visual disturbance
- Breathing difficulty
- Fever or chills
- Difficulty emptying bladder
- Epigastric pain

Baby *has any of:*
- Fast/difficult breathing
- Fever
- Unusually cold
- Stops feeding well
- Less activity than normal
- Whole body becomes yellow

Responsibility for the interpretation and use of the material in this checklist lies with the reader. In no event shall the World Health Organization be liable for damages arising from its use. For more information visit www.who.int/patientsafety.

Completed by ____________________
