# Supplementary material for: Barriers and facilitators for implementing the WHO Safe Childbirth Checklist (SCC) in Mozambique: A qualitative study using the Consolidated Framework for Implementation Research (CFIR)
Source: PLOS Glob Public Health. 2024 Sep 5;4(9):e0003174. doi: 10.1371/journal.pgph.0003174 (PMC11376584; doi:10.1371/journal.pgph.0003174)
Supplement: S2 Text — (DOCX) [file pgph.0003174.s002.docx]

## **A: Clinical Administrator Interview Guide**

The interviewees will receive physical copies of the WHO Safe Childbirth Checklist 2 days before the interview to go through the Checklist content. The investigators will elaborate on the purpose of the Checklist and how to use the Checklist before the interview. The interview will take place after the respondents have consented to participate.

**I.** **Overview of current childbirth practice in the facility (2 questions)**

To begin our conversation, I would like to learn more about the current birth practices in your setting.

Q1. What has been your experience with the current childbirth practice guidelines in your clinics, if any?

a. Can you describe the childbirth practice guidelines which have been used in your setting?

b. What kind of mechanisms have been used to ensure the current guidelines are well followed?

c. How do the current guidelines help with the practices?

Q2. How do you feel about current childbirth practices in your facility?

a. Do you have adequate support, such as financial, material, and human resources, coordination, and management, for childbirth practices?

b. What are the gaps, issues, or problems related to the current childbirth practices?

c. What are the components of the current work routines that could be altered? Why?

d. What are the supplies or equipment that are routinely missing? Why?

**II.** **WHO Safe Childbirth Checklist Characteristics (5 questions)**

First, we will discuss some of your overall impressions of the WHO Safe Childbirth Checklist. We will use “Checklist” to refer to it in the following parts.

Q1. How complicated is the Checklist?

Q2. How does the Checklist compare to the current childbirth practices in your clinic?

Q3. If you would implement the Checklist, what kinds of changes do you think you will make to the Checklist to make it work effectively in your setting?

Q4. What kinds of additional support would help you implement and use the Checklist?

Q5. What additional costs were considered when deciding to implement the Checklist?

**III.** **Inner setting (3 questions)**

Now, we are going to focus on factors within your setting that can influence the Checklist implementation.

Q1. How do you think implementing the Checklist would meet the current needs of childbirth practices in your setting?

a. Probe for how & why.

Q2. How do you think the Checklist could integrate into the existing organizational structures and work procedures in your setting?

a. What kinds of changes will be needed in the hospital to accommodate the implementation of the Checklist?

b. Can you describe how the Checklist will be integrated into current processes?

c. Will the Checklist replace or complement a current program or process?

Q3. What are likely issues or complications that may arise from implementing the Checklist?

a. Probe for costs, resources, training, and workload, asking for examples

**IV.** **Outer setting (2 questions)**

Now, we will explore factors within the broader context that can possibly influence the Checklist implementation. The influence could be positive or negative.

Q1. What kind of local or national performance measures, policies, regulations, or guidelines influenced the implementation of the Checklist?

a. Probe for any reporting and/or monitoring mechanisms

Q2. What kind of financial or other incentives influenced the implementation of the Checklist?

**V.** **Ending Questions (2 questions)**

Here are some ending questions regarding your professional roles and experience.

Q1. Do you have any questions for us?

Q2. Demographic Questions

a. What’s your professional background?

b. What are your specific roles in the hospital?

c. How many years of working experience do you have?

d. How long have you been working in this management position?

e. How old are you?

f. What’s your gender?

## **B: Key Informant Interview Guide**

Ministry of Health Staff Interview Guide

The investigator will present the WHO Safe Childbirth Checklist, explain the purpose of the Checklist and the study, elaborate on how the Checklist should be used, review the Checklist items, and clarify any questions the participant may have regarding the research and the Checklist before beginning the interview. The interview will take place after the respondent has consented to participate.

1. **Overview of current childbirth practice and background in Mozambique (1 question)**

To begin our conversation, I would like to learn more about the current childbirth condition in Mozambique.

Q1. What do you think about current birth practices in Mozambique?

a. Are there components of the current routines that could be altered?

b. What kinds of gaps, issues, or problems could be related to the current childbirth practice?

c. Do birth attendants and the health facilities have adequate support, such as resources, coordination, and management, for the birth practices?

1. **WHO Safe Childbirth Checklist Characteristics (4 questions)**

First, we will discuss some of your overall impressions of the WHO Safe Childbirth Checklist. We will use “Checklist” to refer to it in the following parts.

Q1. How complicated is the Checklist?

a. Which specific Checklist items are more likely to be completed? Which are less likely to be?

b. How does the Safe Childbirth Checklist compare to other alternatives that may have been considered by MOH or that you know about?

Q2. What kinds of changes do you think you will make to the Checklist to make it work effectively in the local health facilities?

c. Are there components that should be altered and should not be altered?

Q3. What kinds of support, such as online resources, education materials, or a toolkit would help the health facilities implement and use the Checklist?

Q4. Implementing the WHO Safe Childbirth Checklist will require leadership commitment and ongoing support, such as coaching, progress measurement, and data feedback, to integrate and sustain the application. What costs were considered when deciding to implement the Checklist from the MOH perspective?

1. **Inner setting (3 questions)**

Now, we are going to focus on factors within the health facility setting that can influence the Checklist implementation.

Q1. How do you think implementing the Checklist would meet the current needs of birth practices in Mozambique?

a. Does implement the Checklist align with the MOH’s goals and priorities regarding the Maternal and Child Health improvement?

Q2. How do you think the Checklist could integrate within the existing organizational structures and workflows in the health facilities?

a. What kinds of changes will be needed to accommodate the implementation of the Checklist?

Q3. What are likely issues or complications that may arise from implementing the Checklist?

1. **Outer setting (3 questions)**

Now, we will explore factors within the larger and external environment that can possibly influence the Checklist implementation. The influence could be positive or negative.

Q1. What kind of local, state, or national performance measures, policies, regulations, or guidelines could influence the implementation of the Checklist?

Q2. What kind of financial or other incentives would influence the implementation of the Checklist?

Q3. How would MOH leverage the relationship of internal and external stakeholders to support the Checklist implementation in the country? The stakeholders might include government officials, local and national administrative staff, NGOs, local health facilities, birth attendants, and patients, etc.

1. **Ending Questions (3 questions)**

Here are some ending questions regarding your professional roles and experience.

Q1. What’s your professional background?

Q2. What are your specific roles in the MOH?

Q3. Do you have any questions for us?

## **C: Birth Attendant Focus Group Discussion Guide**

A Focus Group will include four to five participants. The participants will receive physical copies of the WHO Safe Childbirth Checklist 2 days before the discussion to go through the Checklist content. The investigators will elaborate on the purpose of the Checklist and how to use the Checklist before the discussion. The interview will take place after the respondents have consented to participate.

**I.** **Opening Questions (1 question)**

Introduction of the investigators and participants.

Q1. Do you have questions about the Checklist?

**II.** **Introduction Question (1 question)**

To begin our conversation, we would like to learn

Q1. What has been your experience using the childbirth practice guidelines in your clinics, if any?

a. Can you describe the childbirth practice guidelines which have been used in your settings?

b. What kind of mechanisms have been established to ensure that the current guidelines are well followed?

c. What kind of changes have been made to the current childbirth practice guidelines? If any, why?

d. How did/do the guidelines help with the practices?

**III.** **Transition Question (1 question)**

Q1. What is your experience with the current childbirth practices?

a. What kinds of support did you have for the childbirth practices?

b. What gaps, issues, or problems were related to the current childbirth practice?

Probe for more examples.

**IV.** **Key Questions (8 questions)**

Next, we will discuss some of your overall impressions of the WHO Safe Childbirth Checklist. We will use “Checklist” to refer to it in the following parts.

Q1. How complicated is the Checklist?

Q2. How does the Checklist compare to the current childbirth practices?

Q3. How well does the Checklist fit with existing work routines in your setting?

a. Can you describe how the Checklist will be integrated into current processes?

b. Will the Checklist replace or complement a current program or process?

Q4. How do you think your workplace culture will affect the implementation of the Checklist?

a. How do you feel if you are being asked to make new changes to your current childbirth process?

b. How do you feel if you are being asked to set aside your original work routines?

Q5. What kind of local or national performance measures, policies, or regulations would influence the implementation of the Checklist?

a. Probe for any reporting and/or monitoring mechanisms

Q6. What are likely issues or complications that may arise from implementing the Checklist?

a. Probe for costs, resources, training, and workload, asking for examples

Q7. What kind of additional support would help you implement the Checklist?

Q8. What kinds of changes do you think you will make to the Checklist to make it work effectively in your setting?

**V.** **Ending Questions (4 questions)**

Q1. How do you think implementing the Checklist would meet the current needs of childbirth practices in your setting?

a. Probe for how & why.

Q2. Is there anything else you would like to add?

Q3. Do you have any questions for us?

Q4. Demographic questions

a. What’s your professional background?

b. What are your specific roles in the hospital?

c. How long have you been working in the maternity ward?

d. How old are you?

e. What’s your gender?
